# Antimicrobial Use in Cats in a University Veterinary Hospital in Central Italy: A Retrospective Study

**DOI:** 10.3390/antibiotics13100927

**Published:** 2024-09-27

**Authors:** Matilde Vernaccini, Lucia De Marchi, Angela Briganti, Ilaria Lippi, Veronica Marchetti, Valentina Meucci, Luigi Intorre

**Affiliations:** Veterinary Teaching Hospital, Department of Veterinary Sciences, University of Pisa, 56124 Pisa, Italy; matilde.vernaccini@phd.unipi.it (M.V.); lucia.demarchi@unipi.it (L.D.M.); angela.briganti@unipi.it (A.B.); ilaria.lippi@unipi.it (I.L.); veronica.marchetti@unipi.it (V.M.); luigi.intorre@unipi.it (L.I.)

**Keywords:** cat, antimicrobial prescriptions, internal medicine unit, intensive care unit

## Abstract

**Background:** Monitoring antimicrobial use is crucial for understanding current prescribing practices. Such information helps in establishing stewardship programs for effectively using antimicrobials and combating resistance to them. **Objectives:** This study describes how antimicrobials are prescribed at the Veterinary Teaching Hospital of the University of Pisa and compares how the internal medicine and intensive care units differ in their usage of antimicrobials. **Methods:** The study involved cats that were presented in the units in 2021 and 2022. Antimicrobial prescriptions were obtained via the hospital’s management software (OCIROE). **Results:** In a population of 1164 non-surgical cats with 397 antimicrobials prescribed, the most prescribed ones were amoxicillin–clavulanic acid in the internal medicine unit and ampicillin in the intensive care unit. Twenty-five percent of all antimicrobials were Highest-Priority Critically Important Antimicrobials or Antimicrobial Advice Ad Hoc Expert Group Category B. The oral route was the main route of administration in the internal medicine unit, while parenteral was the most common route used in the intensive care unit. Most antimicrobials were prescribed to treat pathologic conditions affecting the urinary (39%), gastroenteric (21%), respiratory (13%), and integumentary (12%) systems. A diagnosis, accurate dosage, and the use of species-approved medications were recorded in the antimicrobial prescriptions. However, only 11% of these prescriptions were supported by antimicrobial susceptibility tests. **Conclusions:** These results suggest room for improvement, particularly in increasing the use of antimicrobial susceptibility testing to ensure more targeted antimicrobial therapy. Given the importance of antimicrobial resistance and the One Health approach, the study also highlights the need to consider the broader impact of antimicrobial use in animals, including the potential contribution to resistance in bacteria that affect both animal and human health.

## 1. Introduction

The growing concern over antimicrobial resistance (AMR) in companion animals has prompted increased focus on monitoring the use of antimicrobials in these pets, coupled with endeavors to introduce antimicrobial stewardship (AMS) methodologies [1]. Identifying the patterns of antimicrobial prescriptions through continual data collection and analysis is fundamental for improving AMS in veterinary hospitals [2]. Monitoring antimicrobials entails systematic collecting and assessing data on prescriptions [3]. Recognizing and adhering to the judicious use of the most appropriate antimicrobials in veterinary medicine is imperative for safeguarding public health, curtailing the propagation of resistant bacteria, and preserving the effectiveness of antimicrobials for severe infections. The essential role of the veterinarian in ensuring the prudent use of antimicrobials is also emphasized in the new European Regulation 2019/6 on veterinary medicinal products, in which the legislator aimed to strengthen policies to combat antimicrobial resistance. Several articles within the regulation specifically address the use of antimicrobial medicinal products.

The One Health perspective highlights the need for collaborations among practitioners of human and veterinary medicine, environmental sciences, and other relevant fields in order to address the complex issue of AMR. This approach is integral to global initiatives aimed at combating AMR [4,5]. The World Health Organization (WHO) has categorized antimicrobials based on their critical importance to human health, identifying a group known as the Highest-Priority Critically Important Antimicrobials (HPCIAs) [4]. In line with the WHO’s prioritization, the Antimicrobial Advice Ad Hoc Expert Group (AMEG) within the European Union recommends classifying antimicrobials for veterinary use into four categories, from A (avoid) to D (prudence), based on the availability of alternatives in veterinary medicine [6,7]. The aim of this categorization is to support veterinarians in selecting the appropriate antimicrobial for infectious diseases, considering the potential consequences for public health. Additionally, the categorization is used by policymakers to assess the feasibility of applying restrictions on the use of antimicrobial medicines in veterinary medicine to preserve their effectiveness for humans.

Frequent use of antimicrobials within veterinary medicine significantly contributes to the spread of AMR [8,9,10]. Specifically, pet dogs and cats can carry numerous human-related pathogens along with various multi-drug-resistant bacteria, such as methicillin-resistant *Staphylococcus aureus* (MRSA) and beta-lactam antibiotic-resistant Enterobacteriaceae [11,12]. The initial antimicrobial treatment for these patients is empirical and can be adjusted on the basis of bacteriological susceptibility test results [10,13]. Furthermore, conformity to established guidelines or recommendations by AMS programs is often neglected, indicating a general shortfall in AMS adherence and overuse of antimicrobials in treating hospitalized small animals [14].

Consequently, we aim to describe the patterns of antimicrobial prescription in cats at the Veterinary Teaching Hospital of the University of Pisa (VTH) and how antimicrobial usage differs between the internal medicine unit (IMU) and intensive care unit (ICU). By doing so, we hope to contribute to the ongoing surveillance of antimicrobial use in companion animals, which is a pivotal element of AMS.

## 2. Results

### 2.1. Overall Number of Prescriptions

A total of 1403 prescriptions were analyzed for 1164 non-surgical feline patients, with 434 cats from the internal medicine unit (IMU) and 730 cats from the intensive care unit (ICU). The ICU had 248 antimicrobial prescriptions (29%), which was significantly higher than the 97 antimicrobial prescriptions (17.6%) recorded in the IMU (*p* < 0.0001). Overall, there were 853 antimicrobial prescriptions in the ICU and 550 in the IMU. Additionally, a total of 286 (72.3%) antimicrobials were prescribed in the ICU, which was significantly higher than the 111 antimicrobials prescribed (27.7%) in the IMU (*p* < 0.0001). In total, 397 antimicrobials were prescribed across both units. Prescriptions with incomplete information, accounting for 1.3% of the total, were excluded from the analysis.

A total of 11 different antimicrobials from seven different classes were prescribed. The most used classes were aminopenicillins (43%), a fixed combination of amoxicillin–clavulanic acid (24%), and fluoroquinolones (24%), while each of the other classes accounted for less than 4% of the total number of prescriptions.

Specifically, the combinations of amoxicillin–clavulanic acid and ampicillin were the most frequently prescribed antimicrobials in the IMU and ICU, respectively (*p* < 0.0001), constituting ≈60% of all antimicrobials used in each respective unit (Table 1). Enrofloxacin was the next most common prescription in both units (Table 1).

Of the 397 antimicrobials examined, a total of 101 fell under the category of HPCIAs or B AMEG, encompassing 94 fluoroquinolones (enrofloxacin, marbofloxacin, and ofloxacin) and seven third-generation cephalosporins (cefovecin), and they were used more commonly in the ICU than in the IMU.

Antimicrobials were administered as the sole therapy (including fixed-dose combinations) in 314 instances (87%), whereas in 51 cases (13%), antimicrobials were used in empirical combinations, predominantly in the ICU (76%).

The most prevalent empirical combinations in the IMU were the use of amoxicillin–clavulanic acid with enrofloxacin (75%). Within the ICU, combining ampicillin with either enrofloxacin (49%) or marbofloxacin (18%) was common practice (Table 2).

The administration of antimicrobials through the oral route was predominantly used in the IMU and accounted for 95.4% of all prescribed antimicrobials. Conversely, in the ICU, the parenteral route was more common, representing 91.7% of the total number of antimicrobials prescribed. The topical route was less frequent and was mainly in the form of eye drops.

The majority (84%) of antimicrobials were prescribed for pathological conditions affecting four out of ten of the considered systems: urinary (39%), gastroenteric (21%), respiratory (13%), and integumentary (12%) (Table 3). The range of antimicrobial classes used for treating conditions affecting these systems varied from four (respiratory) to six (gastroenteric) (Table 3). Amoxicillin–clavulanic acid in the IMU and ampicillin in the ICU were the most frequently prescribed antimicrobials to treat the pathological conditions affecting these four systems. Fluoroquinolones were primarily used for urinary conditions, constituting 37% of the antimicrobials prescribed for this purpose (44% in the IMU, and 32% in the ICU). These three classes together accounted for 97% of the prescribed antimicrobials.

### 2.2. Appropriate Antimicrobial Prescribing Patterns

For both the IMU and ICU, diagnoses were available for all antimicrobial prescriptions. Additionally, all the antimicrobials prescribed were approved for use in cats and adhered to the specified dose range and duration of therapy as reported in the manufacturer’s information leaflet (Table 4).

A total of 37 ASTs (antimicrobial susceptibility tests) were recorded, equating to an 11% sample submission rate out of the total number of antimicrobial prescriptions. Notably, the submission rate for ASTs was higher in the IMU, at 15%, compared to 9% in the ICU. When antimicrobials classified as HPCIAs or within the B category of the AMEG classification were prescribed, ASTs were conducted on 24 occasions, representing 5% and 19% of the total number of antimicrobial prescriptions in the IMU and ICU, respectively (Table 4).

## 3. Discussion

This research consists of a comprehensive two-year evaluation (2021-2022) of antimicrobial prescription practices for feline patients within the IMU and ICU at the University of Pisa’s VTH. This research is crucial for monitoring antimicrobial usage in companion animals, and thus the effective implementation of AMS programs.

Several limitations of the study need highlighting due to its retrospective design, which focused on a review of the medical records of antimicrobial prescriptions. This approach limits the understanding of the prescription decisions, which could have been influenced by treatments initiated by referring veterinarians before the hospital visit. Despite these limitations, the study results provide valuable insights for implementing AMS programs by understanding (i) to what extent the veterinarians involved adhered to prescribing guidelines and (ii) the full context of treatment decisions.

The antimicrobial prescription rate among our patient cohort was 25%, which closely aligns with the findings of several other surveys, where antimicrobials accounted for 10-25% of cat prescriptions [15,16]. The total number of antimicrobial prescriptions was notably higher in the ICU compared to the IMU. This discrepancy likely stems from the fact that patients in the ICU often require more intensive therapeutic interventions. In contrast, patients in the IMU are typically involved in less acute and less frequently suspected infectious conditions, where the need for antimicrobials is generally less frequent [17,18].

In our investigation, in parallel with observations from comparable veterinary teaching hospitals, antimicrobial prescriptions in felines predominantly targeted pathological conditions impacting the urinary, gastrointestinal, respiratory, and dermatological systems [16,19]. Notably, aminopenicillins, potentiated penicillins, and fluoroquinolones were found to be the principal antimicrobial drug classes prescribed, together accounting for 97% of all antimicrobial treatments. This distribution suggests a uniformity in the choice of antimicrobials across various classes. Specifically, the fixed-dose combination of amoxicillin–clavulanate and ampicillin was the most common treatment employed for treating pathological conditions of the urinary, gastrointestinal, respiratory, and integumentary systems in the IMU and ICU, respectively. This is to some extent in line with the trend of antimicrobial use reported by Robbins et al. [19], in which the commonly prescribed antimicrobials were amoxicillin/clavulanate, metronidazole, and ampicillin/sulbactam, with significant use for integumentary, gastrointestinal, and respiratory diseases.

The present research underscores the crucial role of amoxicillin–clavulanic acid as the primary antimicrobial choice in the IMU, following selection criteria outlined in guidelines for the responsible use of antimicrobials [20,21,22]. This is likely due to its recognized safety, cost-effectiveness, and broad-spectrum efficacy. Additionally, the availability of an oral formulation makes it more convenient for home administration by pet owners, further supporting its widespread adoption in veterinary practices [18,23].

In the ICU, the decision to use ampicillin as the primary antimicrobial is likely due to its broad-spectrum coverage and versatility, including the option for intravenous administration [23]. This underscores the effectiveness, safety, and cost-efficiency of ampicillin, making it a favored option for immediate and broad-ranging antimicrobial intervention in a critical care setting [4].

The frequent prescription of fluoroquinolones in veterinary practice, particularly for urinary infections, is largely attributed to their broad coverage against Gram-negative organisms, while minimizing the risk of nephrotoxicity associated with aminoglycosides [19,20]. Enrofloxacin, a commonly used fluoroquinolone, is highly effective in treating a range of infections in dogs and cats, including those affecting the respiratory, urinary, gastrointestinal, and integumentary systems [24]. Although the use of enrofloxacin aligns with established therapeutic guidelines, it necessitates careful justification, especially for infections resistant to other antimicrobials as determined by susceptibility testing. However, the implications of quinolone use extend beyond the immediate veterinary context. The concern surrounding their use in dogs and cats is linked to broader issues of antimicrobial resistance (AMR) that impact human health. Even though specific quinolones like enrofloxacin might not be used in humans, their application in animals can still contribute to the development of resistance patterns that have significant repercussions for human health. This connection underscores the need for a comprehensive understanding of how veterinary practices influence AMR. To tackle this issue, scientific research has examined various aspects of antimicrobial resistance. Reviews on this topic frequently emphasize that the use of antibiotics, including quinolones, in animals can promote resistance that may impact humans [25]. Additionally, the One Health approach, which explores the link between veterinary and human health, offers valuable perspectives on how veterinary practices, including the use of quinolones, affect human health. In conclusion, while fluoroquinolones such as enrofloxacin are important in veterinary medicine, their use must be carefully regulated to reduce the risk of fostering antimicrobial resistance. Grasping the broader implications of resistance patterns, as outlined in the scientific literature, is crucial for protecting both animal and human health. The widespread prescription of these drugs has created significant selective pressure, leading to the emergence of strains with reduced susceptibility. Consequently, caution is crucial because enrofloxacin can alter the gut microbiome and increase the risk of bacterial resistance [26]. This thus highlights that the extensive use of this antimicrobial family has generated sufficient selection pressure in healthy animals, even without antimicrobial exposure, to harbor fluoroquinolone-resistant strains in their guts.

The relatively low usage of third-generation cephalosporins, despite the convenience and high owner compliance offered by cefovecin, highlights the importance of opting for alternative antimicrobials from an antimicrobial stewardship perspective. The differences in administration practices observed between the IMU and ICU units in this study likely reflect the different therapeutic management approaches in these settings. Hospitalized patients in the ICU often require immediate and intensive treatment, leading to a preference for intravenous administration to ensure rapid therapeutic effects. Conversely, in the IMU unit, where patients might not be as acutely ill, oral administration is favored for its ease and convenience, aligning with long-term management strategies for outpatient care [23].

The use of empirical antimicrobial combinations revealed in our study, although infrequent (13% of prescriptions), primarily involved the combination of fluoroquinolones with amoxicillin, either alone or boosted with clavulanic acid, and targeting pathologic urinary and gastrointestinal system conditions. Despite the limited reliance on ASTs, these empirical combinations are recognized within treatment protocols, justifying their use under specific circumstances. Such scenarios include complex medical and polymicrobial conditions, where these established combinations can provide the essential broad coverage for effective treatment.

In other scenarios, the use of empirical combinations appears unnecessary and often serves the purpose of achieving broad coverage without a precise diagnosis. This approach, while based on established treatment protocols, underscores the need for careful consideration and justification when using empirical antimicrobial combinations, especially in terms of antimicrobial stewardship principles.

In our study, antimicrobial prescriptions were supported by a clinical diagnosis, adherence to recommended dosage and duration of therapy as per the product information leaflet, and the use of drugs approved for the target species. The strict avoidance of off-label antimicrobial use is particularly significant, addressing common concerns in veterinary medicine regarding the off-label application of antimicrobials, especially those critical to human health. These concerns typically involve unwarranted use, incorrect dosing, or improper administration routes, which could lead to ineffective antimicrobial usage, thereby posing a risk to both animal and public health by potentially facilitating the spread of antimicrobial resistance.

In our study, only 11% of antimicrobial prescriptions were supported by AST results, and 24% for prescriptions involving HPCIAs or B AMEG antimicrobials, where ASTs are strongly recommended. The infrequent use of ASTs, also reported in other studies [27], is partly due to the fact that most antimicrobial prescriptions are given in ICU settings, where the management times for suspected sepsis are often not compatible with those required for a culture test. However, this represents a significant deviation from prudent use guidelines, suggesting the need for further interventions to improve the integration of ASTs in monitoring and documenting bacterial susceptibility in veterinary practices [13,19].

## 4. Materials and Methods

### 4.1. Data Collection

This study included cats that received at least one antimicrobial prescription and were examined between 1 January 2021 and 31 December 2022 in the IMU and ICU of the Veterinary Teaching Hospital at the University of Pisa, Italy. Prescriptions were the means by which drugs are dispensed both by community pharmacies and the VTH pharmacy. Prescriptions were issued as electronic prescriptions in accordance with current Italian regulations when medicines were dispensed by community pharmacies, while they were registered in a specific module of the VTH management software (OCIROE) when dispensed by the VTH pharmacy. IMU visits included patients receiving either general or specialist consultations that were discharged within 24 h of their presentation. ICU visits included critical patients temporarily hospitalized for diagnostic investigation or therapeutic management that were discharged and directly handed back to the owner after treatment. Cats undergoing surgery were not included in the study due to the prevalent prophylactic use of antimicrobials.

### 4.2. Data Analysis

Medical records: All medical records were manually reviewed, and the data extracted were provided as Excel files exported from the OCIROE software. Each medical record comprised the animal identification number, date and reason for the clinical evaluation, signalment, weight of the animal upon admission, clinical diagnosis, and antimicrobial prescriptions.

Clinical diagnosis was based on the evaluation of the patient’s clinical signs, medical history, physical examination, and laboratory tests. Clinical diagnoses associated with an antimicrobial prescription were categorized by circulatory, gastroenteric, urinary, lymphatic, muscular, ophthalmic, auditory, respiratory, integumentary, and neurological systems. Medical records with incomplete information were excluded from the study.

Prescriptions: Each prescription included the medicinal product, dosage, duration of treatment, route of administration, and frequency of administration. Each antimicrobial prescription could include one or more antimicrobials.

Medicinal products: Products containing two or more antimicrobials were defined as fixed-dose combinations and categorized into a distinct class [28]. The simultaneous administration of two or more medicinal products containing antimicrobials was defined as an empirical combination. Antimicrobials were described according to the class, route of administration, and condition treated. Prescriptions of antimicrobials classified as HPCIAs by the WHO ranking or as AMEG categories A/B were also evaluated [4].

Antimicrobial susceptibility tests (ASTs): The susceptibility of bacterial isolates was evaluated via broth microdilution tests [23].

Assessment of appropriate antimicrobial prescribing patterns: Antimicrobial prescriptions were considered to reflect appropriate prescribing patterns when there was a clinical diagnosis available, access to relevant antimicrobial susceptibility tests (ASTs), use of medications approved for the specific species, and adherence to recommended dosages as outlined in the medication information leaflet [20,29]. A dosage within ±10% of the recommended dose was deemed acceptable. Adjustment of the dosages were performed according to the patient’s weight, clinical condition, and route of administration.

### 4.3. Statistical Analyses

Frequency distributions from 2021 to 2022 between IMU and ICU cats were tested by Fisher’s exact test for (I) general data on medical consultation and prescriptions, (II) changes in the use of the antimicrobial classes, (III) antimicrobial prescriptions in monotherapy or empirical combination, (IV) distribution of antimicrobial prescriptions by route of administration, and (V) indications for total prescribed antimicrobials by body system categories. Values of *p* ≤ 0.05 were considered statistically significant. All the statistical analyses were performed using Prism 7^®^ (GraphPad Software Inc., San Diego, CA, USA).

## 5. Conclusions

This study found that the antimicrobial prescription rate among the patient population was 25%, with variations between the ICU and IMU. The most commonly prescribed classes of antimicrobials were aminopenicillins, potentiated penicillins, and fluoroquinolones, with amoxicillin–clavulanic acid and ampicillin being the most frequently used agents for various conditions. Differences in prescribing practices between the ICU and IMU were observed, potentially influenced by variations in patient management and therapeutic approaches. The results of this study can only acknowledge what emerges from prescriptions, highlighting whether prescriptions fit in terms of prudent and rational use, with the belief that identifying antimicrobial prescription patterns through data collection and analysis is fundamental for the future implementation of AMS in veterinary hospitals. Despite efforts by veterinarians in our hospital to adhere to prudent use principles, the study identified some areas of concern. Notably, it highlighted the use of antimicrobials in empirical combinations, including those classified as Highest-Priority Critically Important Antimicrobials (HPCIAs) by the WHO or as category B AMEG. While these combinations are part of recognized treatment protocols, their use may not always be justified. Furthermore, antimicrobial susceptibility tests (ASTs) were underutilized, with only a small percentage of prescriptions supported by ASTs. This underuse, although consistent with findings from other studies, is particularly concerning in cases involving HPCIAs or AMEG category B antimicrobials and represents a significant deviation not only from guidelines for prudent antibiotic use but also from the measures aimed at combating resistance that the legislator intended to strengthen through the new European Regulation 2019/6. Overall, our findings underscore the importance of ongoing monitoring and surveillance of antimicrobial use in companion animals to ensure prudent use and reduce the risks of antimicrobial resistance. The excessive or inappropriate use of quinolones observed in this study should also be interpreted within the context of human health, given the potential cross-resistance issues and broader impacts on human healthcare. Efforts to enhance AST use and promote rational prescribing practices are essential, and they must align with the One Health approach to address the broader implications of antimicrobial use, including environmental impacts and the contribution to resistance in bacteria that affect animal and human health.

## Figures and Tables

**Table 1:** Antimicrobials prescribed in the IMU and ICU from 2021 to 2022.

	IMU *n* (%)	ICU *n* (%)	Total	*p*-Value
AMINOGLYCOSIDES	3 (2.7)	1 (0.3)	4	=0.09
Tobramycin *	3	1		
CEPHALOSPORINS	3 (2.7)	10 (3.4)	13	>0.99
Cephalexin	2	-		
Cefazolin	-	4		
Cefovecin	1	6		
FIXED-DOSE COMBINATION	63 (56.7)	34 (11.9)	97	<0.0001
Amoxicillin–Clavulanic Acid	63	34		
FLUOROQUINOLONES	30 (27.0)	64 (22.3)	94	=0.32
Enrofloxacin	22	45		
Marbofloxacin	7	19		
Ofloxacin *	1	-		
LINCOSAMIDES	2 (1.8)	1 (0.3)	3	=0.23
Clindamycin	2	1		
PENICILLINS	0 (0.0)	171 (59.7)	171	<0.0001
Ampicillin	-	171		
TETRACYCLINES	10 (9.0)	5 (1.7)	15	<0.01
Doxycycline	10	5		
Total	111 (100)	286 (100)	397	>0.99

* Used for topical application.

**Table 2:** Empirical antimicrobial prescriptions in the IMU and ICU in 2021–2022.

Combinations	IMU *n* (%)	ICU *n* (%)	Total *n* (%)
Amoxicillin–Clavulanic Acid + Enrofloxacin	9 (75.0)	5 (12.8)	14
Ampicillin + Marbofloxacin	-	7 (17.9)	7
Ampicillin + Enrofloxacin	-	19 (48.7)	19
Others *	3 (25)	8 (20)	11
Total	12 (100)	39 (100)	51

* Empirical combinations with number of prescriptions <4.

**Table 3:** Prescribed antimicrobials by circulatory, gastroenteric, urinary, lymphatic, muscular, ophthalmic, auditory, respiratory, integumentary, and neurological systems in the IMU and ICU from 2021 to 2022.

	AMN		CEF		LINC		PEN		FX-DC		QUI		TTR		TOT
	IMU	ICU	IMU	ICU	IMU	ICU	IMU	ICU	IMU	ICU	IMU	ICU	IMU	ICU	*n* (%)
Circulatory	-	-	-	-	-	-	-	3	-	-	-	-	-	-	3 (0.7)
Gastroenteric	-	-	-	1	1	-	-	44	13	5	3	13	2	-	82 (20.6)
Urinary	-	-	-	2	-	-	-	59	29	7	24	32	1	-	154 (38.8)
Lymphatic	-	-	-	-	-	-	-	2	1	1	-	1	1	-	6 (1.5)
Muscular	-	-	-	4	-	-	-	17	6	5	3	4	-	-	39 (9.8)
Ophthalmic	3	1	-	-	-	-	-	-	2	-	-	-	3	2	11 (2.8)
Auditory	-	-	-	-	-	-	-	-	1	1	-	-	-	-	2 (0.5)
Respiratory	-	-	1	2	-	-	-	28	6	4	1	7	-	3	52 (13.1)
Integumentary	-	-	-	1	-	1	-	20	9	11	-	5	-	-	47 (11.8)
Neurological	-	-	-	-	-	-	-	1	-	-	-	-	-	-	1 (0.2)
Total	3	1	1	9	1	1	-	174	67	34	31	62	7	5	397 (100)

AMN: aminoglycosides; CEF: cephalosporins; LINC: lincosamides; PEN: penicillins; FX-DC: fixed-dose combination; QUI: fluoroquinolones; TTR: tetracyclines; TOT: total.

**Table 4:** Antimicrobial prescribing patterns in the IMU and the ICU from 2021 to 2022.

Antimicrobial Prescribing Patterns			
	IMU *n* (%)	ICU *n* (%)	Total *n* (%)
*Availability of:*			
- Diagnosis ^a^	97 (100)	248 (100)	345
- Antimicrobial susceptibility tests ^a^	16 (15)	21 (9)	37 (11)
- Antimicrobial susceptibility tests ^b^	5 (5)	19 (19)	24 (24)
*Agreement with:*			
- Use of a product approved for species	97 (100)	248 (100)	345
- Appropriate the dose range	97 (100)	248 (100)	345
- Appropriate duration of treatment	97 (100)	248 (100)	345

^a^ Out of 345 prescribed antimicrobials; ^b^ out of 101 prescribed HPCIAs/B AMEG.

## Data Availability

No new data were created or analyzed in this study. Data sharing is not applicable to this article.
